# Incorporating biological information in sparse principal component analysis with application to genomic data

**DOI:** 10.1186/s12859-017-1740-7

**Published:** 2017-07-11

**Authors:** Ziyi Li, Sandra E. Safo, Qi Long

**Affiliations:** 1 Department of Biostatistics and Bioinformatics, Emory University, 1518 Clifton Road, Atlanta, 30322 GA USA; 2 Department of Biostatistics, Epidemiology and Informatics, Perelman School of Medicine, University of Pennsylvania, 423 Guardian Drive, Philadelphia, 19104 PA USA

**Keywords:** Principal component analysis, Sparsity, Structural information, Genomic data

## Abstract

**Background:**

Sparse principal component analysis (PCA) is a popular tool for dimensionality reduction, pattern recognition, and visualization of high dimensional data. It has been recognized that complex biological mechanisms occur through concerted relationships of multiple genes working in networks that are often represented by graphs. Recent work has shown that incorporating such biological information improves feature selection and prediction performance in regression analysis, but there has been limited work on extending this approach to PCA. In this article, we propose two new sparse PCA methods called Fused and Grouped sparse PCA that enable incorporation of prior biological information in variable selection.

**Results:**

Our simulation studies suggest that, compared to existing sparse PCA methods, the proposed methods achieve higher sensitivity and specificity when the graph structure is correctly specified, and are fairly robust to misspecified graph structures. Application to a glioblastoma gene expression dataset identified pathways that are suggested in the literature to be related with glioblastoma.

**Conclusions:**

The proposed sparse PCA methods Fused and Grouped sparse PCA can effectively incorporate prior biological information in variable selection, leading to improved feature selection and more interpretable principal component loadings and potentially providing insights on molecular underpinnings of complex diseases.

**Electronic supplementary material:**

The online version of this article (doi:10.1186/s12859-017-1740-7) contains supplementary material, which is available to authorized users.

## Background

A central problem in high-dimensional genomic research is to identify a subset of genes and pathways that can help explain the total variation in high-dimensional genomic data with as little loss of information as possible. Principal component analysis (PCA) [1] is a popular multivariate analysis method which seeks to concentrate the total information in data with a few linear combinations of the available data, making it an appropriate tool for dimensionality reduction, data analysis, and visualization in genomic research. Despite its popularity, the traditional PCA is often difficult to interpret as the principal component loadings are linear combinations of all available variables, the number of which can be very large for genomic data. It is therefore desirable to obtain interpretable principal components that use a subset of the available data to deal with the problem of interpretability of principal component loadings.

Several alternatives to PCA have been proposed in the literature, most of which constrain the size of non-zero principal component loadings. An ad hoc approach sets the absolute value of loadings that are smaller than a threshold to zero. Though simple to understand, this approach has been shown to be misleading in the sense that magnitude of loadings is not the only factor to determine the importance of variables in a linear combination [2]. Truncating PCs by loadings may result in quite different PCs explaining much smaller variation compared with the original PCs. Other approaches regularize the loadings to ensure that some are exactly zero, which implies that the corresponding variables are unimportant in explaining the total variation in the data. For instance, Jolliffe et al. [3] proposed the SCotLass method that constrains the loadings with a lasso penalty, but their optimization problem is nonconvex, which is difficult to solve and does not guarantee convergence to a global solution. Zou et al. [4] proposed a convex sparse PCA method (SPCA) that reformulates the PCA problem as a regression problem and imposes elastic net penalty on the PC loadings. Witten and Tibshirani [5] also proposed the penalized matrix decomposition (PMD) that approximates the data with its spectral decomposition and imposes a lasso penalty on the right singular vectors, i.e., the principal component loadings.

Although the aforementioned methods can effectively produce sparse principal component coefficients, their main limitation is that they are purely data driven and do not exploit available biological information such as gene networks. It has been recognized that complex biological mechanisms occur through concerted relationships of multiple genes working together in pathways. Recent work [6, 7] has demonstrated in the regression setting that utilizing prior biological information among variables can improve variable selection and prediction and help gain a better understanding of analysis results. It is therefore desirable to conduct PCA with incorporation of known structural information. Allen et al. [8] proposed a generalized least-square matrix decomposition framework for PCA that incorporates known structure of noise and generate sparse solutions. Although this method can flexibly account for noise structure in data, they do not utilize prior biological information, and do not consider the relationships among the signal variables in PCA. Jenatton et al. [9] proposed a structured sparse PCA method that considers correlations among groups of variables and imposes a penalty similar to group lasso on the principal component loadings, but their method does not take into account the complex interactions among variables within a group. In this article, we proposed two new sparse PCA methods called Fused and Grouped sparse PCA that enable incorporation of prior biological information in PCA. The methods will allow for identification of genes and pathways. We generalize fused lasso [10] and utilize *L*_*γ*_ norm [7] to achieve automatic variable selection and simultaneously account for complex relationships within pathways.

Our work makes several contributions. To the best of our knowledge, this is the first attempt to impose both sparsity and smoothing penalties on principal component loadings to encourage the selection of variables that are connected in a network. Although Jenatton et al. [9] and Shiga and Mamitsuka [11] incorporated group information of variables when generating sparse PC solutions, they did not consider how variables are connected in each group. Our method considers not only the group information, but also any interaction structure of variables within a group. By utilizing the existing biological structure in the data, we are able to obtain sparse principal components that are more interpretable and may shed light on the underlying complex mechanisms in the data. We also develop an efficient algorithm that can handle high-dimensional problems. Simulation studies suggest that the methods have higher sensitivity and specificity in detecting true signals and ignoring noise variables, and are quite effective in improving the performance of sparse PCA methods when the graph structure is correctly specified. In addition, the proposed methods are robust to misspecified graph structure.

The remainder of the paper is organized as follows. In “Methods” section, we present methods and algorithms for the proposed sparse PCA. In “Results” section, we conduct simulation studies to assess the performance of our methods in comparison with several existing sparse PCA methods. In “Analysis of Glioblastoma data” section, we apply the proposed methods to data from a glioblastoma brain multiform study. We conclude with some discussion remarks in “Discussions” section.

## Methods

Suppose that we have a random *n*×*p* matrix $\mathbf {X} = (\mathbf {x}_{1},\ldots,\mathbf {x}_{p}), \mathbf {x} \in \mathfrak {R}^{n}$. We also assume that the predictors are centered to have column means zero. The network informaton for the *p* variables in **X** is represented by a weighted undirected graph $\mathcal {G}=(C,E,W)$, where *C* is the set of nodes corresponding to the *p* features, *E*={*i*∼*j*} is the set of edges indicating that features *i* and *j* are associated in a biologically meaningful way, and *W* includes the weight of each node. For node *i*, denote by *d*_*i*_ its degree, i.e., the number of nodes that are directly connected to node *i* and by *w*_*i*_=*f*(*d*_*i*_) its weight which can depend on *d*_*i*_. Our goal is to obtain sparse PCA loadings while utilizing available structural information $\mathcal {G}$ in PCA. Our approach to the sparse PCA problem relies on the eigenvalue formulation of PCA, and for completeness sake, we briefly review the classical and sparse PCA problems.

### Standard and sparse principal component analysis

Classical PCA finds projections $\boldsymbol {\alpha } \in \mathfrak {R}^{p}$ such that the variance of the standardized linear combination **X** ***α*** is maximized. Mathematically, the first principal component loading ***α*** solves the optimization problem

1 $$  \max_{\boldsymbol{\alpha}\ne {\mathbf{0}}} {\boldsymbol{\alpha}}^{\text{T}}\mathbf{X}^{\text{T}}\mathbf{X}{\boldsymbol{\alpha}} ~~~\text{subject to}~~ {\boldsymbol{\alpha}}^{\text{T}}{\boldsymbol{\alpha}} =1.  $$

For subsequent principal components, additional constraints are added to ensure that they are uncorrelated with previous principal components, so that each principal component axis captures different information in the data. Generally, for the *r*th PC, we have the optimization problem

2 $$\begin{array}{@{}rcl@{}}  \underset{\boldsymbol{\alpha}_{r} \ne \mathbf{0}}{\text{max}} &{\boldsymbol{\alpha}}_{r}^{\text{T}}\mathbf{X}^{\text{T}}\mathbf{X} {\boldsymbol{\alpha}}_{r} ~~~\\ & \text{subject to}~~ {\boldsymbol{\alpha}}_{r}^{\text{T}}{\boldsymbol{\alpha}}_{r} =1, {\boldsymbol{\alpha}}_{s}^{\text{T}}{\boldsymbol{\alpha}}_{r} =0 \ \\ & \forall s<r,~~r=2,\dots,q\ll \min(p,n-1). \ \end{array} $$

Using Lagrangian multipliers, one can show that problem (2) results in the eigenvalue problem

3 $$\begin{array}{@{}rcl@{}} \mathbf{X}^{\text{T}}\mathbf{X}{\boldsymbol{\alpha}}= {\lambda}{\boldsymbol{\alpha}}. \end{array} $$

Then the *r*th principal component loadings of **X** is the *r*th eigenvector that corresponds to the *r*th eigenvalue $\tilde {\lambda }_{1} \geq \cdots \geq \tilde {\lambda }_{r} \geq \cdots \geq 0$ of the sample covariance matrix **X** ^T^ **X**. Of note, the magnitude, *α*_*rk*_ of each principal component loading $\tilde {\boldsymbol {\alpha }}_{r}=[\alpha _{r1},\ldots,\alpha _{rk},\ldots,\alpha _{rp}]$ represents the importance of the *k*th variable to the *r*th principal component, and these are typically nonzero. When *p*≫*n*, interpreting the principal components is a difficult task because the principal components are linear combinations of all variables. Thus for high-dimensional data, a certain type of regularization that ensures that some variables have negligible or no effect on the *r*th principal component is warranted to yield interpretable principal components.

To achieve sparsity of the principal component loadings while incorporating structural information $\mathcal {G}$, we utilize ideas in Safo and Ahn [12] which is motivated by the Dantzig Selector for sparse estimation in regression problems. Specifically, we bound a modified version of the eigenvalue difference in (3) with a *l*_*∞*_ norm while minimizing a structured-sparsity inducing penalty of the principal component loadings:

$$\begin{array}{*{20}l} \underset{{\boldsymbol{\alpha}}\ne \mathbf{0}}{\text{min}}\mathcal{P}({\boldsymbol{\alpha}}, \tau)~&\text{subject to}~ \|\mathbf{X}^{\text{T}}\mathbf{X}\tilde{\boldsymbol{\alpha}}_{r}- {\tilde{\lambda}_{r}}{\boldsymbol{\alpha}} \|_{\infty} \leq\tau \\ &\text{and} ~~ {\mathbf{A}}_{r-1}^{\text{T}}{\boldsymbol{\alpha}} = 0. \ \end{array} $$

Here, for a random vector $\mathbf {z} \in \mathfrak {R}^{p}$, ∥**z**∥_*∞*_ is the *l*_*∞*_ norm defined as max_1≤*i*≤*p*_|*z*_*i*_|, *τ*>0 is a tuning parameter that controls how many of the coefficients in the principal component loadings will be exactly zero. In addition, $\mathbf {A} = [\hat {\boldsymbol {\alpha }}_{1},\ldots,\hat {\boldsymbol {\alpha }}_{s}] ~\forall s < r$ is a concatenation of the previous sparse PCA solutions $\hat {\boldsymbol {\alpha }}_{s}$, and $\tilde {\boldsymbol {\alpha }}_{r}$ is the nonsparse *r*th PCA loading, which is the eigenvector corresponding to the *r*th largest eigenvalue $\hat {\lambda }_{r}$ of **X** ^T^ **X**.

There are a few advantages of this new formulation over the standard formulation for PCA. First, the objective function $\mathcal {P}(\boldsymbol {\alpha }, \tau)$ can easily incorporate the prior information about the PC loadings, for example, the structural information of variables. Second, this optimization problem can be easily solved by any off-the-shelf optimization software given $\mathcal {P}(\boldsymbol {\alpha }, \tau)$ is a convex function, e.g. CVX in Matlab. In the next sections, we introduce sparse PCA methods that utilize the network information $\mathcal {G}$ in **X**.

### Grouped sparse PCA

The first approach we propose is the grouped sparse PCA, similar in spirit with Pan et al. [7]. Utilizing the graph structure $\mathcal {G}$, we propose the following structured sparse PCA criterion for the *r*th principal component loading:

4 $$\begin{array}{@{}rcl@{}} \underset{\boldsymbol{\alpha} \ne \mathbf{0}}{{\min}}\!\! &\left\{(1-\eta)\sum_{i \sim j}\left(\frac{|\alpha_{i}|^{\gamma}}{w_{i}} + \frac{|\alpha_{j}|^{\gamma}}{w_{j}}\right)^{1/\gamma} + \eta\sum_{d_{i}=0} |\alpha_{i}| \right\} \\ &\text{subject to} \quad \|\mathbf{X}^{\text{T}}\mathbf{X}\tilde{\boldsymbol{\alpha}_{r}}- {\tilde{\lambda}_{r}}\boldsymbol{\alpha} \|_{\infty} \leq\tau ~~ \text{and} ~~ {\mathbf{A}}_{r-1}^{\text{T}}\boldsymbol{\alpha} = 0, \ \end{array} $$

where ∥·∥_*∞*_ is the *l*_*∞*_ norm, *τ*>0 is a tuning parameter, *γ*>1 and 0<*η*<1 are fixed, $\mathbf A_{r-1}=(\hat {\alpha }_{1},\hat {\alpha }_{2},\ldots,\hat {\alpha }_{r-1})$ is the matrix constituted of *r*−1 structured sparse PC loadings, and $\tilde {\boldsymbol {\alpha }}_{r}$ is the *r*th nonsparse PC loading vector, which is the eigenvector corresponding to the *r*th largest eigenvalue of **X** ^T^ **X**.

The first term in the objective function (4) is the weighted grouped penalty of Pan et al. [7], which induces grouped variable selection. The penalty encourages both *α*_*i*_ and *α*_*j*_ to be equal to zero simultaneously, suggesting that two neighboring genes in a network are more likely to participate in the same biological process simultaneously. The second term in the objective function induces sparsity in selection of singletons that are not connected to any other variables in the network. The tuning parameter *τ* enforces some coefficients of the principal components to be exactly zero with larger values encouraging more sparsity. The selection of *τ* is usually data-driven, and is discussed in section 2.4. The optimization problem is convex in ***α*** and can be solved with any off the shelf convex optimization package such as the CVX package [13] in Matlab.

### Fused sparse PCA

The second structured sparse PCA is the Fused sparse PCA, which generalizes fused lasso [10] to account for complex interactions within a pathway. Utilizing the graph structure $\mathcal {G}$, we propose the following structured sparse PCA for the *r*th principal component loading:

5 $$\begin{array}{@{}rcl@{}}  \underset{\boldsymbol{\alpha} \ne \mathbf{0}}{{\min}}\!\!\! & \left\{(1-\eta)\sum_{i\sim j}\left|\frac{\alpha_{i}}{w_{i}}- \frac{\alpha_{j}}{w_{j}}\right| + \eta\sum_{d_{i}=0}|\alpha_{j}|\right\} \\ &~\text{subject to} \quad \|\mathbf{X}^{\text{T}}\mathbf{X}\tilde{\boldsymbol{\alpha}}_{r}- {\tilde{\lambda}_{r}}{\boldsymbol{\alpha}} \|_{\infty} \leq\tau~~ \text{and} ~~ {\mathbf{A}}_{r-1}^{\text{T}}\boldsymbol{\alpha} = 0 \ \end{array} $$

where *τ*>0 is tuning parameters, 0≤*η*≤1 is fixed, $\mathbf {A}_{r-1}=(\hat {\alpha }_{1},\hat {\alpha }_{2},\ldots,\hat {\alpha }_{r-1})$ is the matrix constituted of *r*−1 structured sparse PC loadings, and $\tilde {\boldsymbol {\alpha }}_{r}$ is the *r*th nonsparse PC loading vector. This penalty is a combination of weighted *l*_1_ penalty on variables that are connected in the network and *l*_1_ penalty on singletons that are not connected to any genes in the network. The first term in the objective function (5) is the fused structured penalty that encourages the difference between variable pairs that are connected in the network to be small and hence the variables to be selected together.

This penalty is similar to some existing penalties, but different in a number of ways. First, it is similar to the fused lasso—both attempt to smooth the coefficients that are connected in $\mathcal {G}$. However, the fused lasso does not utilize prior biological information. Instead, it uses a data-driven clustering approach to order the variables that are correlated and imposes *l*_1_ penalty on the difference between coefficients of adjacent variables. It also does not weight neighboring features, which may allow one to enforce various prior relationships among features. Second, the Fused sparse penalty is also similar but different to the network constrained penalty of Li and Li [6]. Their penalty $ \eta _{1}\sum _{j}|\alpha _{j}| + \eta _{2}\sum _{i\sim j}\left (\frac {\alpha _{i}}{w_{i}}- \frac {\alpha _{j}}{w_{j}}\right)^{2}$ uses the *l*_2_ norm and it has been shown that this does not produce sparse solutions, where sparsity refers to variables that are connected in a network. In other words, it does not encourage grouped selection of variables in the network [7]. Also, the additional tuning parameter *η*_2_ increases computational costs for very large *p* since it requires solving a graph-constrained regression problem with dimension (*n*+*p*)×*p*.

The two proposed methods differ in how the structural information is incorporated in the PCA problem. Grouped sPCA is dependent on *γ* in the *L*_*γ*_ norm and have different sparsity solution in the PC loadings for different *γ*. Unlike the Fused sPCA, the weights in the Grouped sPCA allow for two neighboring nodes to have opposite effects, which may be relevant in some biological process. However, in the Fused sPCA, it is easy to understand that the *l*_1_ norm difference of connected pairs allows variables that are connected or behave similarly to be close together, which is not so intuitive in the Grouped sPCA.

### Algorithms

We present two algorithms for the proposed structured sparse PCA methods. Algorithm 1 obtains the *r*th principal component loading vector for a fixed tuning parameter *τ*. Algorithm 2 provides a data driven approach for selecting the optimal tuning parameter value *τ* from a range of values. The normalization in step (3) of Algorithm 1 eases interpretation, and usually facilitates a visual comparison of the coefficients. Once the principal component loading vector is obtained, the coefficients (in absolute value) can be ranked to assess the contribution of the variables to a given PC. Both our methods require the data to be centered (column-centered for a *n*×*p* matrix) so that PCA can be conducted on covariance matrix. If the variables are measured on different scales or on a common scale with widely differing ranges, it is recommended to center and scale the variables to have unit variance before implementing the proposed methods.





Algorithm 1 is developed to obtain *r* PC loading vectors. For the best *r*, we can introduce tuning parameter selection in step (2) using, for example cross validation to maximize the total variance explained by the *r*th principal component, with the smallest *r* explaining some proportion of variance explained selected as the optimal *r*th principal component. This would add extra layer of complexity to the tuning parameter selection, however.

The tuning parameters *τ*=(*τ*_1_,…*τ*_*r*_) control the model complexity and their optimal values need to be selected. We use Bayesian information criterion (BIC) [8] and implement Algorithm 2 to select *τ* that yields a better rank *r* approximation to the test data. Compared with using cross-validation to select best tuning parameters, BIC can be computationally more efficient, especially for large datasets. The selection of the other tuning parameters in our experiments are described as follows. We fix *η*=0.5 for an equal likelihood of selecting networks and singletons. Since Pan et al. [7] chose gamma=2 and 8 and showed that these two gamma values achieved good performance, we fix *γ*=2 for both the simulation study and the real data analysis and we also compare in a subset of simulations *γ*=2 and *γ*=8 (see Additional file 1: Tables S1 and S2) to assess whether the results are robust to the gamma value. We set *w*_*i*_ and *w*_*j*_ as the degree of each node following the suggestion in Pan et al. [7]. Our paper seeks to develop methods for estimating sparse principal components, as such it is not the focus of the paper to investigate principled approaches for selecting the number of principal components that will be used in subsequent analyses. We use the top two principal components in both our simulation study and the real data analysis. In practice, some ad-hoc approaches, such as choosing the top K PCs with more than 80% variation explained, can be used.

## Results

We conduct numerical studies including simiulations and real data analysis to assess the performance of the proposed methods in comparison with several existing sparse PCA methods. We consider two simulation settings that differ by the proportions of variation explained by the first two PCs. In the first setting, the first two PCs explain 6% of the total variation which indicates that true signals in the data are weak. In the second setting, the first two PC’s explain 30% of the total variation in the data, representing a case where signals are strong. Within each setting, we consider the dimensions *p*=500 and *p*=10,000, and also consider two scenarios that differ by the graph structure $\mathcal {G}$ for the proposed methods.

### Simulation settings

Let **X** be a *n*×*p* matrix and let **G**_0_ be the true covariance matrix used to generate **X**. Let $\mathcal {G}_{0}$ be the corresponding graph structure. The true covariance matrix **G**_0_ is partitioned as

$$\begin{array}{@{}rcl@{}} \mathbf{G}_{0} =\left(\begin{array}{cc} \mathbf{G}_{00} & \mathbf{0} \\ \mathbf{0} & \nu \times \mathbf{I}_{p-36} \ \end{array} \right), \end{array} $$

where **G**_00_ is block diagonal with ten blocks each of size 18 for *p*=500 and size 250 for *p*=10,000, and between block correlation 0. We set the variance of variables in the first two blocks to be 1, and 0.3 for the remaining eight blocks. In addition, we set the correlation of a main and connecting variable to be 0.9 for the first two blocks and 0.2 for the other blocks. Meanwhile, we let the correlation *ρ*_*ik*_∼*Uniform*(0.7,0.8),*i*≠*k* *and* *i,k*≥2 for the first two blocks, and *ρ*_*ik*_∼*Uniform*(0,0.2),*i*≠*k* *and* *i,k*≥2 for the other blocks. This type of covariance matrix **G**_0_ suggests that data structure is determined by ten underlying subnetworks, where the first two PCs of the first two subnetworks are mostly important in detecting signals in the data. In other words, in both settings, the true PCs has 36 important variables and *p*−36 noise variables when *p*=500, and *p*=500 important variables and *p*−500 noise variables for *p*=10,000. We note that by changing the value of *ν*, we control the proportions of variation explained by the first two PCs. The *ν* values we used in both simulation settings are presented in Additional file 1: Table S3. For each setting, we specify *n*=100, and simulate **X** from multivariate normal distribution with mean **0** and variance **G**_0_.

**Fig. 1:**
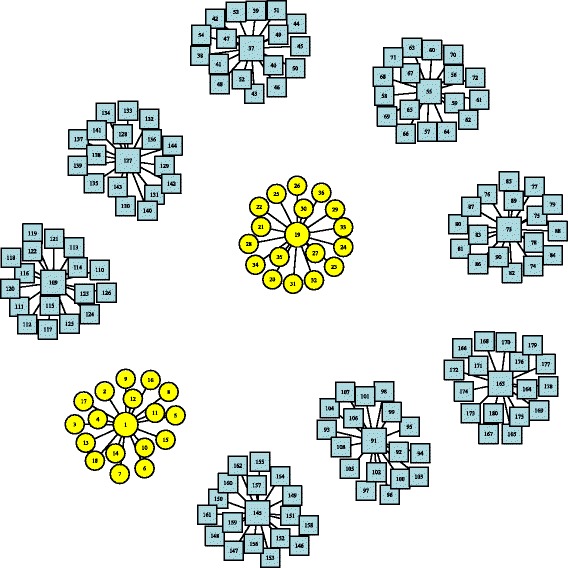
Network structure of simulated data: Correctly specified graph. Variables in *circle* represent signals, and *square* represent noise. ($\mathcal {G}=\mathcal {G}_{0}$)

For each setting and dimension, we consider two scenarios that differ by the graph structure $\mathcal {G}$ specified in the proposed sPCA methods. In the first scenario, the graph structure is correctly specified, that is $\mathcal {G}= \mathcal {G}_{0}$. This corresponds to the situation where all true structural information are available in $\mathcal {G}$ so that $\mathcal {G}$ is informative. The resulting network includes 500 variables and 170 edges between each main variable and connecting variable when *p* equals 500 (or 10,000 variables and 2490 edges when *p* equals 10,000), i.e., *E*={*i*∼*j*|*i*,*j*=1,⋯,180} in $\mathcal {G}$ when *p* equals 500 (or *E*={*i*∼*j*|*i, j*=1,⋯,2,500} in $\mathcal {G}$ when *p* equals 10,000). Figure 1 is a graph of the network $\mathcal {G}$ used in Fused and Grouped sPCA when network information is correctly specified.

In the second scenario, the graph structure is randomly generated and does not capture the true information in the data. The resulting network includes a total of 170 random edges when *p* equals 500 (or 2490 edges when *p* equals 10,000). We first generate a *p*×*p* matrix with each element from *U*(0,1) distribution. The elements with values more than an arbitrary cutoff 0.95 are saved as candidates for random edges by considering their row numbers and column numbers are connected nodes. We then choose a random subset with size 170 (or 2490) as the noninformative structure. It is possible that few random edges have overlaps with informative edges, but most of them are still noises. This setting assesses the performance of the proposed methods in cases where the structural information is uninformative and sheds light on robustness of the proposed methods. Additional file 1: Figure S1 shows the graph structure for randomly specified edges.

*Performance Metrics* We compare the proposed methods Grouped PCA and Fused PCA to the traditional PCA [1], SPCA [4] and SPC [14]. We implement SPCA and SPC using the R-packages *elasticnet* and *PMA* respectively. We evaluate the performance of the methods using the following criteria.

- *Reconstruction error*: $||\mathbf {X}_{test}\mathbf {A}\mathbf {A}^{T} -\mathbf {X}_{test}\hat {\mathbf {A}}\hat {\mathbf {A}}^{\text {T}}||^{2}_{F}$, where **A**=(***α***_1_ ***α***_2_) are the true PC loadings and $\hat {\mathbf {A}}=(\hat {\boldsymbol {\alpha }}_{1} ~\hat {\boldsymbol {\alpha }}_{2})$ are the estimated PC loadings. This criterion tests the methods ability to approximate the testing data reconstructed using only the first two PC loadings.
- *Estimation error*: $||\mathbf {A}\mathbf {A}^{T} -\hat {\mathbf {A}}\hat {\mathbf {A}}^{\text {T}}||^{2}_{F}$. This criterion tests the methods ability to estimate the linear subspace spanned by the true PC loadings [15], with a smaller estimate preferred.
- *Selectivity*: We also test the methods ability to select the right variables while ignoring noise variables using sensitivity and specificity which are defined as $ Sensitivity = \frac {\#\ \text {of}\ \text {True}\ \text {Positive}}{\#\ \text {of}\ \text {True}\ \text {Positive} + \#\ \text {of}\ \text {False}\ \text {Negative}}, $ $ Specificity = \frac {\#\ \text {of}\ \text {True}\ \text {Negative}}{\#\ \text {of}\ \text {True}\ \text {Negative} + \#\ \text {of}\ \text {False}\ \text {Positive}}. $ Sensitivity and specificity capture the accuracy of estimated PC loadings with high values indicating better performance.
- *Proportion of variance explained*: The fourth comparison criterion is the proportion of variation explained in the testing and training data sets by the first two PC loadings, which is defined as $ \frac {\hat {\boldsymbol {\alpha }}^{\text {T}}\mathbf {X}\mathbf {X}^{\text {T}}\hat {\boldsymbol {\alpha }}}{\text {trace}(\mathbf {X}\mathbf {X}^{\text {T}})}, $ where **X** is either the centered training or testing data set, and $\hat {\text {\boldmath {\({\alpha }\)}}}$ is the estimated first or second PC.

### Simulation results

Table 1 shows the performance of the methods for the first setting where the first two PCs explain only 6% of the total variation in the data. We observe that the proposed methods are competitive for *p*=500 and even more so when *p*=10,000. In particular, Grouped sPCA has smaller reconstruction and estimation errors when the graph structure is correctly specified and even when the graph structure is uninformative. On the other hand, Fused sPCA shows a suboptimal performance in comparison to Grouped sPCA, yet better or competitive performance when compared to the traditional PCA and SPCA for correctly specified graph structure and mis-specified graph structure. In terms of sensitivity and specificity, we observe that both Grouped sPCA and more especially Fused sPCA are better in detecting signals even when the graph structure is mis-specified, while Grouped sPCA is more competitive at not selecting noise variables. We also notice that both Grouped sPCA and Fused sPCA have good performance in proportions of cumulative variation explained compared with existing sparse PCA methods, especially compared with SPCA. In Table 2 where the first two PC’s explain 30% of the total variation in the data, we observe a similar performance of the proposed methods.

**Table 1:** Simulation results of setting 1

Method	RE	EE	Sensitivity		Specificity		cPVE	
			1stPC	2ndPC	1stPC	2ndPC	1stPC	2ndPC
*P* = 500								
PCA	31 (9e-1)	1.1 (3e-2)	1.0	1.0	0.0	0.0	4.3e-2 (2e-3)	8.2e-2 (2e-3)
SPCA	34 (3)	1.2 (1e-1)	0.54	0.50	0.95	0.90	2.0e-2 (2e-3)	4.0e-2 (4e-3)
SPC	16 (8)	0.57 (3e-1)	0.57	0.60	0.98	1.0	2.8e-2 (3e-3)	5.5e-2 (6e-3)
Biological information correctly specified								
Fused sPCA	25 (6)	0.90 (2e-1)	1.0	1.0	0.73	0.70	2.9e-2 (4e-3)	5.1e-2 (7e-3)
Grouped sPCA	8.0 (6)	0.29 (2e-1)	0.81	0.80	0.97	1.0	3.2e-2 (2e-3)	6.0e-2 (3e-3)
Biological information randomly specified								
Fused sPCA	32 (4)	1.1 (2e-1)	0.95	1.0	0.51	0.51	3.0e-2 (4e-3)	5.2e-2 (7e-3)
Grouped sPCA	9.1 (6)	0.33 (2e-1)	0.81	0.80	0.97	1.0	3.2e-2 (2e-3)	5.9e-2 (3e-3)
*P* = 10,000								
PCA	112 (3)	1.3 (2e-2)	1.0	1.0	0.0	0.0	2.6e-2 (1e-3)	5.0e-2 (1e-3)
SPCA	160 (4)	1.9 (3e-2)	0.15	0.15	0.99	0.99	2.3e-3 (5e-4)	4.5e-3 (7e-4)
SPC	172 (4)	2.0 (8e-3)	0.01	0.01	1.0	1.0	1.7e-4 (1e-4)	3.4e-4 (3e-4)
Biological information correctly specified								
Fused sPCA	81 (50)	0.94 (0.5)	0.62	0.55	0.99	0.99	1.2e-2 (6e-3)	2.2e-2 (1e-2)
Grouped sPCA	54 (40)	0.62 (0.4)	0.62	0.58	0.99	1.0	1.4e-2 (3e-3)	2.6e-2 (6e-3)
Biological information randomly specified								
Fused sPCA	140 (30)	1.6 (0.4)	0.60	0.60	0.68	0.68	8.9e-3 (5e-3)	1.6e-2 (1e-2)
Grouped sPCA	58 (40)	0.67 (0.5)	0.59	0.55	0.99	1.0	1.4e-2 (3e-3)	2.6e-2 (7e-2)

Cumulative proportions of variance explained by true PCs are 0.03 for PC 1 and 0.06 for PC 1 and 2. *P*, number of variables. RE, reconstruction error, defined as $||\mathbf {X}_{test}\mathbf {A}\mathbf {A}^{T} -\mathbf {X}_{test}\hat {\mathbf {A}}\hat {\mathbf {A}}^{\text {T}}||^{2}_{F}$, where **A**=(***α***_1_ ***α***_2_). EE, estimation error, defined as $||\mathbf {A}\mathbf {A}^{T} -\hat {\mathbf {A}}\hat {\mathbf {A}}^{\text {T}}||^{2}_{F}$. cPVE, proportions of cumulative variation explained. ·(·), mean(std)

**Table 2:** Simulation results of setting 2

Method	RE	EE	Sensitivity		Specificity		cPVE	
			1stPC	2ndPC	1stPC	2ndPC	1stPC	2ndPC
*P* = 500								
PCA	31 (0.9)	1.1 (3e-2)	1.0	1.0	0.0	0.0	4.3e-2 (2e-3)	8.2e-2 (2e-3)
SPCA	35 (2)	1.3 (9e-2)	0.49	0.50	0.95	1.0	1.9e-2 (3e-3)	3.9e-2 (4e-3)
SPC	15 (7)	0.54 (3e-1)	0.57	0.60	0.98	1.0	2.8e-2 (3e-3)	5.6e-2 (5e-3)
Biological information correctly specified								
Fused sPCA	27 (4)	0.93 (2e-1)	1.0	1.0	0.70	0.70	3.0e-2 (3e-3)	5.3e-2 (5e-3)
Grouped sPCA	7.9 (5)	0.29 (2e-1)	0.80	0.80	0.97	1.0	3.2e-2(2e-3)	6.0e-2 (3e-3)
Biological information randomly specified								
Fused sPCA	32 (5)	1.1 (2e-1)	0.96	1.0	0.52	0.50	2.9e-2 (5e-3)	5.1e-2 (8e-3)
Grouped sPCA	9.2 (6)	0.33 (0.2)	0.79	0.8	0.97	1.0	3.2e-2 (2e-3)	5.9e-2 (4e-3)
*P* = 10,000								
PCA	112 (3)	1.3 (2e-2)	1.0	1.0	0.0	0.0	2.7e-2 (1e-3)	5.0e-2 (1e-3)
SPCA	162 (4)	1.9 (3e-2)	0.16	0.16	1.0	1.0	2.0e-3 (5e-4)	4.0e-3 (8e-4)
SPC	173 (4)	2.0 (5e-3)	5.0e-3	5.0e-3	1.0	1.0	1.6e-4 (1e-4)	3.2e-4 (2e-4)
Biological information correctly specified								
Fused sPCA	77 (40)	0.89 (0.5)	0.65	0.57	0.99	1.0	1.3e-2 (5e-3)	2.3e-2 (9e-3)
Grouped sPCA	46 (30)	0.53 (0.4)	0.65	0.62	0.99	1.0	1.5e-2 (2e-3)	2.8e-2 (5e-3)
Biological information randomly specified								
Fused sPCA	140 (30)	1.6 (0.4)	0.59	0.60	0.68	0.70	9.0e-3 (5e-3)	1.7e-2 (1e-2)
Grouped sPCA	53 (40)	0.61 (0.4)	0.63	0.60	0.99	1.0	1.5e-2 (3e-3)	2.7e-2 (6e-3)

Cumulative proportions of variance explained by true PCs are 0.15 for PC 1 and 0.30 for PC 1 and 2. *P*, number of variables. RE, reconstruction error, defined as $||\mathbf {X}_{test}\mathbf {A}\mathbf {A}^{T} -\mathbf {X}_{test}\hat {\mathbf {A}}\hat {\mathbf {A}}^{\text {T}}||^{2}_{F}$, where **A**=(***α***_1_ ***α***_2_). EE, estimation error, defined as $||\mathbf {A}\mathbf {A}^{T} -\hat {\mathbf {A}}\hat {\mathbf {A}}^{\text {T}}||^{2}_{F}$. cPVE, proportions of cumulative variation explained. ·(·), mean(std)

A comparison between *p*=500 and *p*=10,000 scenarios for both settings indicates that the gain in reconstruction error, estimation error, sensitivity, and proportions of variation explained can be substantial for Grouped sPCA and Fused sPCA compared with the existing sparse PCA methods, as the number of variables increases. This suggests that Grouped sPCA or Fused sPCA can achieve sparse PC loading estimations with higher accuracy, better variable selection, and larger proportion of variation explained, especially when the number of variables is relatively large.

We evaluate the results on different *γ* values. Both Tables 1 and 2 use *γ*=2 and the results of the same settings with *γ*=8 are presented in Additional file 1: Tables S1 and S2. A comparison of Table 1 versus Additional file 1: Table S1 (or Table 2 versus Additional file 1: Table S2) shows very similar results, indicating that the proposed methods are robust to the different selection of *γ* values. We also explore how much the results would be impacted by adding noise structural information in both settings with *P*=500. The results are demonstrated in Additional file 1: Tables S4 and S5. We find that the results by both Fused sPCA and Grouped sPCA worsen a little as expected after adding 170 noise edges. We also find that Grouped sPCA is more robust to noise information than Fused sPCA. After noise informtion is added, Grouped sPCA still has good performance.

### Analysis of Glioblastoma data

We apply the proposed methods to analyze data from a Glioblastoma cancer study. Glioblastoma brain multiform (GBM) is the most common malignant brain tumor and is defined as grade IV astrocytoma by the Whold Health Organization because of its aggressive and malignant nature [16]. The Cancer Genome Atlas Project (TCGA) [17] integratively analyzed genome information of patients with glioblastoma and expanded the knowledge about the pathways and genes that may relate with glioblastoma. In our data analysis, we obtain part of the genomic data from TCGA project for glioblastoma, which is explained in detail by McLendon et al. [17], Verhaak et al. [18], Cooper et al. [19]. This data set contains microarray data of 558 subjects with glioblastoma. The GBM subtype of each subject is also given.

The goal of the analysis is to identify a subset of relevant genes that contribute to the variation in the different GBM subtypes, and also determine how the first two estimated PCs separate these subtypes. For both datasets, we first select 2,000 variables with the largest variation following the data preprocessing procedure in Witten et al. [14]. In the next step, we select patients with subtype *Classical*, *Mesenchymal*, *Neural*, and *Proneural* following the previous work by Verhaak et al. [18] resulting in 481 patients with subtype data. We obtain the gene network information for Fused and Grouped sparse PCA methods from the Kyoto Encyclopedia of Genes and Genomes (KEGG) database [20]. The resulting network has 2000 genes and 1297 edges in the network. We center each variable to have mean 0 and standardize each variable to have variance one.

To justify the structural information we use for the proposed methods, we conduct exploratory analysis using correlation coefficients of gene pairs. We group the gene pairs consisting of the selected 2000 genes into three categories: unconnected gene pairs (two genes that are not in any pathway), direct-connected gene pairs (two genes that have a direct edge connecting them), indirect-connected gene pairs (two genes that belong to the same pathway but do not have a direct edge connecting them) according to the KEGG Pathway information and we use boxplots to demonstrate the correlation coefficients of these three types of gene pairs. Additional file 1: Figure S2 shows the plot of correlation coefficients of gene pairs by their categories. There is a small but clear decreasing trend in correlation coefficients as one moves from direct-connected gene pairs to unconnected gene pairs. This shows that the gene pairs that are directly connected tend to have stronger correlations than those that are indirectly connected or unconnected, thus justifying the validity of pathway information we use in the analysis.

In the analysis, we equally split each data set into training and testing sets, where the training set is used to estimate the optimal tuning parameters via BIC. The plots of BIC values versus tuning parameters for Grouped sPCA and Fused sPCA are shown in Additional file 1: Figure S3. We then apply the optimal parameters on the whole training set to estimate the first two PC loadings $\hat {\boldsymbol {\alpha }}_{i},i=1,2$, and use the testing set to evaluate the estimated loadings using the following two criteria:

$$\begin{array}{*{20}l} \text{Number\ of\ non-zero\ loadings\ of}\\ \hat{\boldsymbol{\alpha}}_{i} = \boldsymbol{\Sigma}_{j=1}^{2000}I\{\hat{\boldsymbol{\alpha}}_{ij}\ne 0\},\quad i=1,2; \end{array} $$

$$\begin{array}{*{20}l} \text{Proportion\ of\ variation\ explained\ by}\\ \hat{\boldsymbol{\alpha}}_{i} = \frac{ \hat{\boldsymbol{\alpha}}_{i}^{\text{T}}\mathbf{X}\hat{\boldsymbol{\alpha}}_{i}}{\text{trace}(\mathbf{X}\mathbf{X}^{T})},\quad i=1,2, \end{array} $$

where **X** is the centered training or testing data matrix. We also obtain the first two PCs $\hat {\boldsymbol {\alpha }}$ by $\hat {\boldsymbol {\alpha }}_{i}=\mathbf {X}\hat {\boldsymbol {\alpha }}_{i}$, *i*=1,2 and determine how well they separate patients with different GBM subtypes using support vector machine (SVM).

Table 3 shows the number of non-zero loadings, the cumulative proportions of variation explained by the first two PC loadings, and the classification results using SVM. We find that SPC and SPCA are more sparse than the Fused sparse PCA and the Grouped sparse PCA. This is consistent with the simulation settings where SPC and SPCA tend to be more sparse and have higher false negatives that result in lower sensitivity. Regarding cumulative proportions of variation explained, we find that the proposed methods explain higher variation in the data, but this may be due to the large number of variables selected. The last column of Table 3 gives the classification results from applying SVM on the testing set using the estimated first two PC loadings. The Fused and Grouped sparse PCA have the highest number of correctly specified subjects. Of the existing methods, PCA and SPCA achieve good performance of separating patients with different subtypes, while SPC has the lowest number of subjects correctly classified.

**Table 3:** Analysis of the GBM data using Kegg Pathway information. cPVE represents proportions of cumulative variation explained

Method	Non-zero Loadings		cPVE		Subjects correctly classified
	1stPC	2ndPC	1stPC	2ndPC	SVM
PCA	2000	2000	0.1955	0.3175	97
SPCA	240	238	0.0333	0.0591	97
SPC	45	59	0.0215	0.0383	67
Fused sPCA	1644	1410	0.1792	0.2787	123
Grouped sPCA	1330	970	0.1731	0.2652	119

We also conduct pathway enrichment analysis using bioinformatics software ToppGene Suite [21]. We take the first PC as an example for illustration. We identify the genes that have non-zero loadings in the first PC from the proposed sparse PCA methods and existing methods, and obtain significantly enriched pathways that are associated with glioblastoma for each method. We seek to identify methods that have more glioblastoma-associated pathways, and whether these overlap. Table 4 shows the Glioblastoma-related pathways found by the proposed methods and existing sparse PCA methods. Among the existing sparse PCA methods, both SPC and SPCA find Spinal Cord Injury pathway. Compared with the existing methods, Fused and Grouped sPCA find a few new Glioblastoma-related pathways: Proteoglycans in cancer, Transcriptional misregulation in cancer, Pathways in cancer, Bladder cancer, and Angiogenesis. These pathways have been demonstrated in existing literatures to be associated with Glioblastoma [22–27]. We do not conduct pathway enrichment analysis with the results of traditional PCA because traditional PCA does not perform any variable selection and automatically select all variables. We also plot the first two PC loadings by Fused and Grouped sPCA in Additional file 1: Figure S4 and the loadings of genes enriched in Glioblastoma-related pathways are highlighted in color. These results indicate that the proposed methods may be more sensitive in detecting disease related signals and thus can identify more biologically important genes.

**Table 4:** Enriched Glioblastoma-related pathways for the genes in first PC by different sPCA methods

Pathway ID	Pathway name	*P*-value	Gene	
			From input	In annotation
Fused sPCA				
739007	Spinal cord injury	7.43E-18	45	112
782000	Proteoglycans in cancer	5.77E-11	55	225
523016	Transcriptional misregulation in cancer	3.31E-7	40	179
83105	Pathways in cancer	3.36E-7	61	327
83115	Bladder cancer	6.10E-6	14	38
Grouped sPCA				
739007	Spinal Cord Injury	1.97E-14	36	112
523016	Transcriptional misregulation in cancer	4.06E-7	34	179
83105	Pathways in cancer	2.58E-5	46	327
P00005	Angiogenesis	4.90E-5	26	150
SPC				
739007	Spinal Cord Injury	1.43E-5	5	112
SPCA				
739007	Spinal Cord Injury	6.46E-5	8	112

## Discussions

In this paper, we propose two novel structured sparse PCA methods. Through extensive simulation studies and an application to Glioblastoma gene expression data, we demonstrate that incorporating known biological information improves the performance of sparse PCA methods. Specifically, our simulation study indicates that the proposed methods can decrease reconstruction and estimation errors, and increase sensitivity and proportions of variation explained, especially when number of variables is large. Compared with Fused sPCA and existing PCA methods, Grouped sPCA achieves the lowest reconstruction error and estimation error for correctly specified and mis-specified graph structure. On the other hand, Fused sPCA has higher sensitivity values. Because we utilize prior biological information, the proposed methods usually have less sparse PC loadings compared with the existing sPCA methods and thus lower specificity. However, there is a trade-off between sparsity and the benefit from extra information. Consistent with the simulations results, the real data analysis demonstrates that the proposed methods generate less sparse PC loadings. However, the classification results show the advantages of incorporating biological information into sparse PCA.

The proposed methods require the structure of variables to be known in advance and specified during analysis. In real data analysis, this task is not trivial and it may take some efforts in searching for a proper variable structure to use. Regarding this, we make the following comments. First of all, many sources of structural information may be available to use including KEGG pathway [20], Panther pathway [28], Human protein reference database [29]. It may be helpful to conduct some exploratory analysis such as Additional file 1: Figure S2 to confirm the need for using biological information. Additional file 1: Figure S2 demonstrates that gene pairs connected in the same pathway generally have higher correlation than gene pairs unconnected in the same pathway, and further than gene pairs in different pathways. Second, our simulation study indicates that even if the structural information is irrelevant as in the biological information randomly specified section, the proposed methods still perform well, especially Grouped sPCA method.

Our proposed methods have some limitations. First, when structural information includes a large number of edges, the proposed methods, particularly, Fused sPCA, may generate PC loadings that include more false positive selections. To solve this problem, one potential approach is to obtain a smaller but more relevant biological structure. Second, the proposed methods, especially Grouped sPCA may be computationally slow in the presence of a large number of edges. Based on our experience with the simulations and the real data set, Fused sPCA is computationally more efficient than Grouped sPCA since we are able to vectorize the penalty for Fused sPCA in the algorithm. Lastly, it has been observed that many studies used gene expression data that are inefficiently and insufficiently pre-processed or normalized, which leads to failure of eliminating technical noise or batch effects [30]. Our proposed methods do not provide steps for pre-processing or normalizing data. The users should adequately pre-process gene expression data to remove potential technical noises and batch effects before applying our methods.

Our structured sparse PCA methods are aimed for estimating sparse PCs and can be considered a dimension reduction technique. Subsequent analyses could use the estimated PCs in a number of different ways. For example, one could use PCs for visualizing gene expression data, clustering, or building prediction model. Following suggestions from a reviewer, we conducted one additional set of simulations to assess the prediction performance of using the top *k* PCs that achieve a certain proportion of total variation explained, and the impact of different threshold values for the proportion of total variation explained. We used a simulation setting similar to Setting 2 in the Simulation section with 100 subject, 500 variables, and 100 simulated datasets. The cumulative proportions of variation explained by the first two PCs are 30%. We generated a binary outcome variable using the first PC through a logistic regression model: *l* *o* *g* *i* *t*(*P* *r*(*Y*_*i*_=1))=0.5+*P* *C*_1*i*_. The simulation results presented in Additional file 1: Table S6 show that Fused sPCA has the highest prediction accuracy among all the sparse PCA methods when 30, 50, and 60% are used as the threshold, consistent with our findings in real data analysis. Also, the prediction accuracy is not very sensitive to the choice of threshold values. Of note, in these simulations, the proportion of total variation explained by all PCs estimated using sparse PCA methods fails to reach 70% for our method and 60% for other methods, which is likely due to regularization/sparsity. It has been reported previously [14, 31] that sparse PCA generates PC solutions that explain smaller proportions of total variation than standard PCA. Future research is needed to investigate more principled approaches for choosing the top *k* PCs in subsequent analysis and to understand why the proportion of total variation explained by all PCs estimated using sparse PCA methods fails to reach certain threshold and potential remedy for this limitation.

Although we apply the proposed methods to analysis of gene expression data, our methods are flexible and general enough to be applied to other data types, such as epi-genomics data discussed in the review paper by Qin et al. [32]. Besides the potential application to other data, some extensions are of potential interest. One may use alternative convex optimization solvers other than the CVX solver in Matlab used in our work, potentially to speed up the computations. In addition, Fused and Grouped sPCA only incorporate the edge information in a graph. As variables are often grouped into pathways, sPCA using hierarchical penalties [33] can be developed to incorporate group membership information in addition to edge information.

## Conclusions

The proposed sparse PCA methods Fused and Grouped sparse PCA can effectively incorporate prior biological information in variable selection, leading to improved feature selection and more interpretable principal component loadings and potentially providing insights on molecular underpinnings of complex diseases.

## Additional file

Additional file 1
**Figure S1.** Network structure of simulated data : Randomly specified graph ($\mathcal {G}$). **Figure S2.** Correlation of gene pairs by relationship types. **Figure S3.** BIC value by tuning parameter with GBM microarray data. X-axis is tuning parameter, y-axis is BIC value. **Figure S4.** Loading plots of the first two PCs by Fused and Grouped sPCA. Colored points are genes enriched in Glioblastoma related pathways found by the proposed methods but not found by existing methods. **Table S1.** Simulation results of Setting 1 when *γ*equals 8. **Table S2.** Simulation results of Setting 2 when *γ* equals 8. **Table S3.** *ν* value used in the simulation settings. **Table S4.** Simulation results of Setting 1 when extra noise edges are added to structural information. **Table S5.** Simulation results of Setting 2 when extra noise edges are added to structural information. **Table S6.** Prediction accuracy using the PCs of PCA-based methods. ·(·) represents mean(sd). (PDF 1270 kb)

## Abbreviations

GBM: Glioblastoma brain multiform
KEGG: Kyoto encyclopedia of genes and genomes
PCA: Principal component analysis
PMD: Penalized matrix decomposition
TCGA: The cancer genome atlas project
