# Supplementary material for: Incorporating biological information in sparse principal component analysis with application to genomic data
Source: BMC Bioinformatics. 2017 Jul 11;18:332. doi: 10.1186/s12859-017-1740-7 (PMC5504598; doi:10.1186/s12859-017-1740-7)
Supplement: Additional file 1 — Figure S1. Network structure of simulated data : Randomly specified graph ($\mathcal {G}$G). Figure S2. Correlation of gene pairs by relationship types. Figure S3. BIC value by tuning parameter with GBM microarray data. X-axis is tuning parameter, y-axis is BIC value. Figure S4. Loading plots of the first two PCs by Fused and Grouped sPCA. Colored points are genes enriched in Glioblastoma related pathways found by the proposed methods but not found by existing methods. Table S1. Simulation results of Setting 1 when γequals 8. Table S2. Simulation results of Setting 2 when γ equals 8. Table S3. ν value used in the simulation settings. Table S4. Simulation results of Setting 1 when extra noise edges are added to structural information. Table S5. Simulation results of Setting 2 when extra noise edges are added to structural information. Table S6. Prediction accuracy using the PCs of PCA-based methods. ·(·) represents mean(sd). (PDF 1270 kb) [file 12859_2017_1740_MOESM1_ESM.pdf]

Incorporating Biological Information in Sparse Principal Component Analysis with Application to Genomic Data

Supplementary materials

Ziyi Li[1], Sandra Safo[1] and Qi Long[2]

[1]Department of Biostatistics and Bioinformatics, Emory University, Atlanta, 30322, USA.
[2]Department of Biostatistics and Epidemiology, Perelman School of Medicine, University of Pennsylvania, 423 Guardian Drive, 19104 Philadelphia, PA, USA.

June 16, 2017

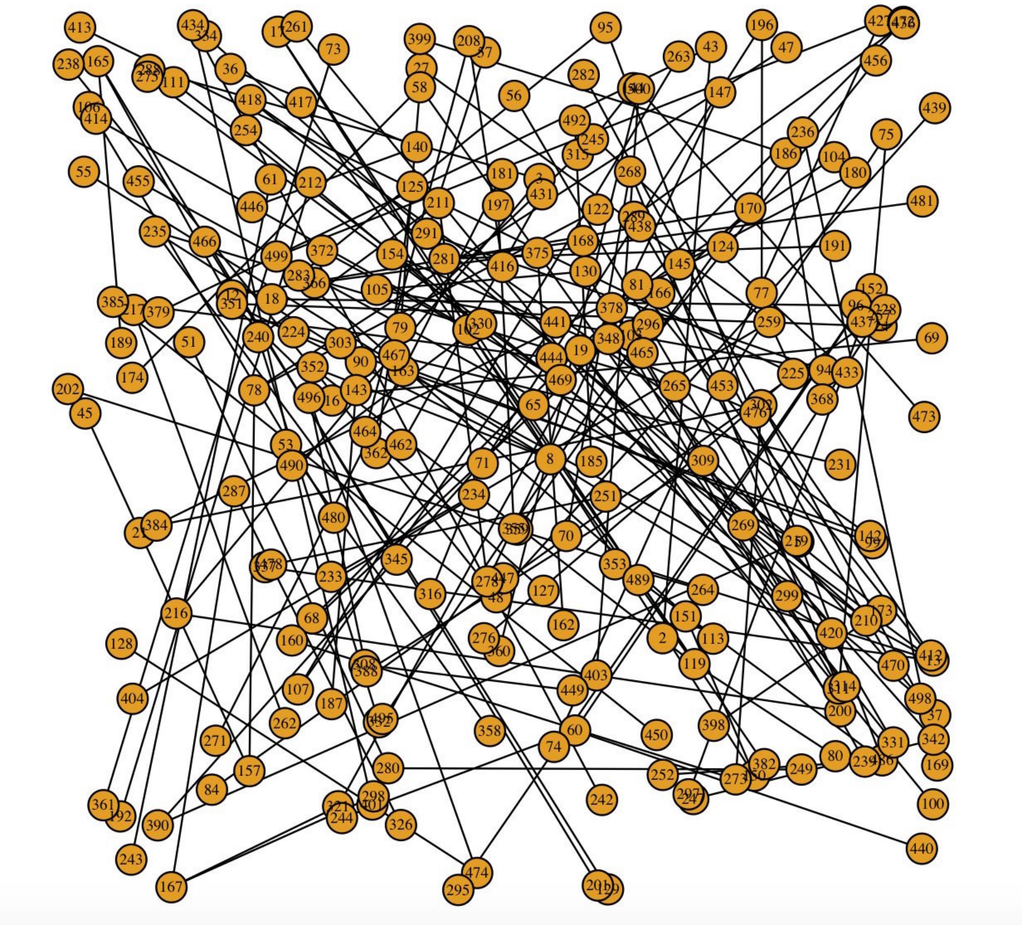

Figure S1: Network structure of simulated data : Randomly specified graph ($\mathcal{G}$)

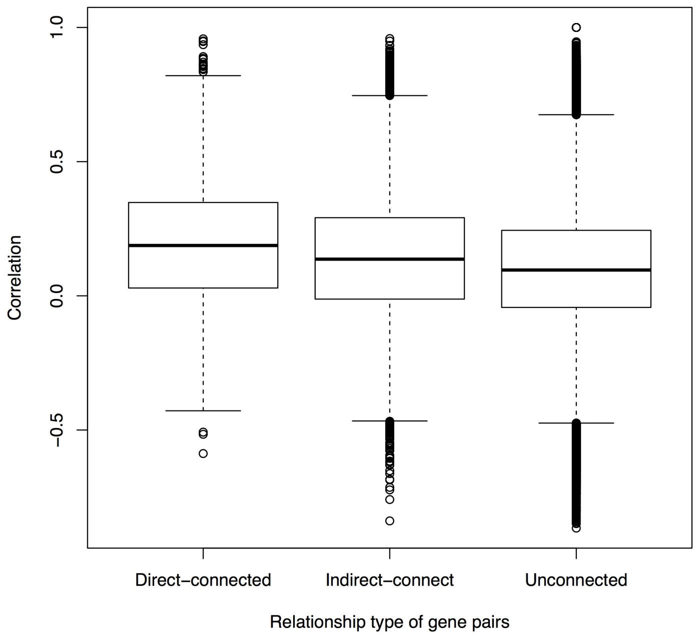

Figure S2: Correlation of gene pairs by relationship types

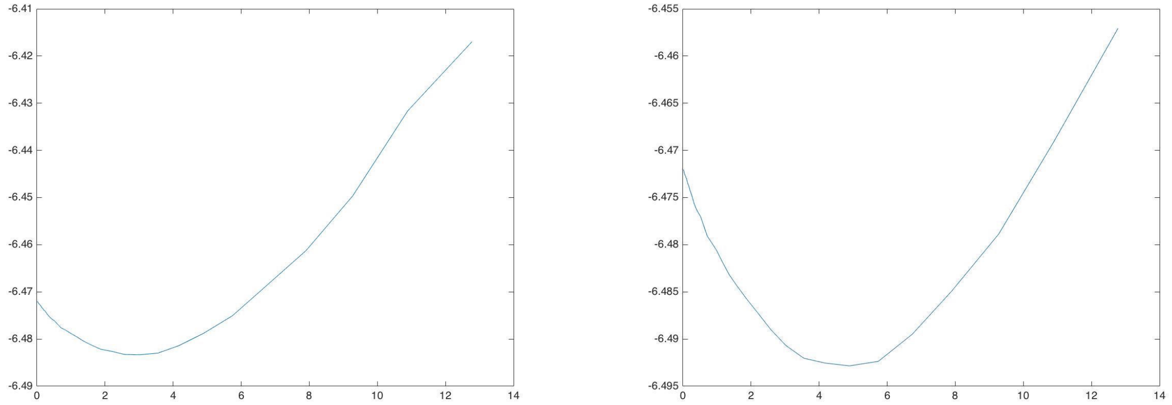

(a) Fused BIC value by tuning parameter.

(b) Grouped BIC value by tuning parameter.

Figure S3: BIC value by tuning parameter with GBM microarray data. X-axis is tuning parameter, y-axis is BIC value.

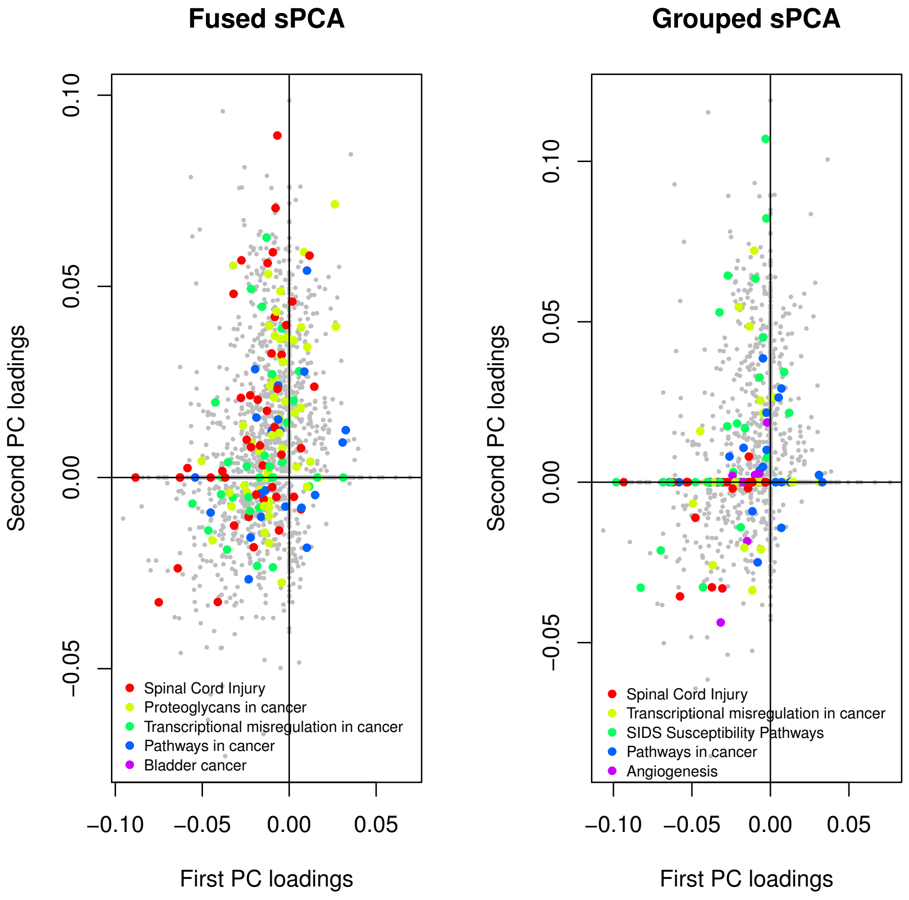

Figure S4: Loading plots of the first two PCs by Fused and Grouped sPCA. Colored points are genes enriched in Glioblastoma related pathways found by the proposed methods but not found by existing methods.

Table S1: Simulation results of Setting 1 when γ equals 8. Cumulative proportions of variance explained by true PCs are 0.03 for PC 1 and 0.06 for PC 1 and 2. P, number of variables. RE, reconstruction error, defined as $||\mathbf{X}_{test}\mathbf{A}\mathbf{A}^T - \mathbf{X}_{test}\hat{\mathbf{A}}\hat{\mathbf{A}}^{\mathrm{T}}||_F^2$, where $\mathbf{A} = (\boldsymbol{\alpha}_1\ \boldsymbol{\alpha}_2)$. EE, estimation error, defined as $||\mathbf{A}\mathbf{A}^T - \hat{\mathbf{A}}\hat{\mathbf{A}}^{\mathrm{T}}||_F^2$. cPVE, proportions of cumulative variation explained. $\cdot(\cdot)$, mean(std).

Method	RE	EE	Sensitivity		Specificity		cPVE	
			1stPC	2ndPC	1stPC	2ndPC	1stPC	2ndPC
**P = 500**								
PCA	31 (9e-1)	1.1 (3e-2)	1.0	1.0	0.0	0.0	4.3e-2 (2e-3)	8.2e-2 (2e-3)
SPCA	34 (3)	1.2 (1e-1)	0.54	0.50	0.95	0.90	2.0e-2 (2e-3)	4.0e-2 (4e-3)
SPC	16 (8)	0.57 (3e-1)	0.57	0.60	0.98	1.0	2.8e-2 (3e-3)	5.5e-2 (6e-3)
biological information correctly specified								
Fused sPCA	25 (5)	0.88 (0.2)	1	1	0.72	0.7	2.9e-2 (3e-3)	5.2e-2 (6e-3)
Grouped sPCA	8.3 (6)	0.3 (0.2)	0.8	0.8	0.97	1	3.2e-2 (2e-3)	5.9e-2 (4e-3)
biological information randomly specified								
Fused sPCA	31 (4)	1.1 (0.1)	0.96	1	0.5	0.5	3e-2 (4e-3)	5.4e-2 (6e-3)
Grouped sPCA	9.2 (6)	0.33 (0.2)	0.8	0.8	0.97	1	3.2e-2 (2e-3)	5.9e-2 (3e-3)
**P = 10,000**								
PCA	112 (3)	1.3 (2e-2)	1.0	1.0	0.0	0.0	2.6e-2 (1e-3)	5.0e-2 (1e-3)
SPCA	160 (4)	1.9 (3e-2)	0.15	0.15	0.99	0.99	2.3e-3 (5e-4)	4.5e-3 (7e-4)
SPC	172 (4)	2.0 (8e-3)	0.01	0.01	1.0	1.0	1.7e-4 (1e-4)	3.4e-4 (3e-4)
biological information correctly specified								
Fused sPCA	97 (60)	1.1 (0.7)	0.45	0.4	0.99	1	9.5e-3 (7e-3)	1.7e-2 (1e-2)
Grouped sPCA	63 (50)	0.73 (0.6)	0.54	0.5	0.99	1	1.3e-2 (4e-3)	2.4e-2 (8e-3)
biological information randomly specified								
Fused sPCA	140 (30)	1.6 (0.4)	0.54	0.5	0.68	0.7	7.9e-3 (5e-3)	1.5e-2 (9e-3)
Grouped sPCA	63 (50)	0.73 (0.6)	0.54	0.5	0.99	1	1.3e-2 (4e-3)	2.4e-2 (8e-3)

Table S2: Simulation results of Setting 2 when γ equals 8. Cumulative proportions of variance explained by true PCs are 0.15 for PC 1 and 0.30 for PC 1 and 2. P, number of variables. RE, reconstruction error, defined as $||\mathbf{X}_{test}\mathbf{A}\mathbf{A}^T - \mathbf{X}_{test}\hat{\mathbf{A}}\hat{\mathbf{A}}^{\mathrm{T}}||_F^2$, where $\mathbf{A} = (\boldsymbol{\alpha}_1\ \boldsymbol{\alpha}_2)$. EE, estimation error, defined as $||\mathbf{A}\mathbf{A}^T - \hat{\mathbf{A}}\hat{\mathbf{A}}^{\mathrm{T}}||_F^2$. cPVE, proportions of cumulative variation explained. $\cdot(\cdot)$, mean(std).

Method	RE	EE	Sensitivity		Specificity		cPVE	
			1stPC	2ndPC	1stPC	2ndPC	1stPC	2ndPC
**P = 500**								
PCA	31 (0.9)	1.1 (3e-2)	1.0	1.0	0.0	0.0	4.3e-2 (2e-3)	8.2e-2 (2e-3)
SPCA	35 (2)	1.3 (9e-2)	0.49	0.50	0.95	1.0	1.9e-2 (3e-3)	3.9e-2 (4e-3)
SPC	15 (7)	0.54 (3e-1)	0.57	0.60	0.98	1.0	2.8e-2 (3e-3)	5.6e-2 (5e-3)
biological information correctly specified								
Fused sPCA	26 (4)	0.92 (0.2)	1	1	0.7	0.7	3e-2 (3e-3)	5.4e-2 (5e-3)
Grouped sPCA	7.8 (5)	0.28 (0.2)	0.8	0.8	0.97	1	3.2e-2 (2e-3)	6e-2 (3e-3)
biological information randomly specified								
Fused sPCA	31 (4)	1.1 (0.1)	0.96	1	0.49	0.5	3.1e-2 (3e-3)	5.4e-2 (6e-3)
Grouped sPCA	9.3 (6)	0.33 (0.2)	0.8	0.8	0.97	1	3.2e-2 (2e-3)	5.9e-2 (3e-3)
**P = 10,000**								
PCA	112 (3)	1.3 (2e-2)	1.0	1.0	0.0	0.0	2.7e-2 (1e-3)	5.0e-2 (1e-3)
SPCA	162 (4)	1.9 (3e-2)	0.16	0.16	1.0	1.0	2.0e-3 (5e-4)	4.0e-3 (8e-4)
SPC	173 (4)	2.0 (5e-3)	5.0e-3	5.0e-3	1.0	1.0	1.6e-4 (1e-4)	3.2e-4 (2e-4)
biological information correctly specified								
Fused sPCA	94 (60)	1.1 (0.7)	0.47	0.4	0.99	1	1e-2 (7e-3)	1.9e-2 (1e-2)
Grouped sPCA	59 (50)	0.67 (0.6)	0.56	0.5	0.99	1	1.4e-2 (5e-3)	2.5e-2 (9e-3)
biological information randomly specified								
Fused sPCA	140 (30)	1.6 (0.4)	0.54	0.6	0.68	0.7	8.2e-3 (5e-3)	1.5e-2 (1e-2)
Grouped sPCA	59 (50)	0.68 (0.6)	0.56	0.5	0.99	1	1.4e-2 (5e-3)	2.5e-2 (9e-3)

Table S3: ν value used in the simulation settings.

	First two PCs explain 3% and 6%	First two PCs explain 15% and 30%
$p = 500$	1.19	0.026
$p = 10,000$	0.27	0.005

Table S4: Simulation results of Setting 1 when extra noise edges are added to structural information. Cumulative proportions of variance explained by true PCs are 0.03 for PC 1 and 0.06 for PC 1 and 2. P, number of variables. RE, reconstruction error, defined as $||\mathbf{X}_{test}\mathbf{A}\mathbf{A}^T - \mathbf{X}_{test}\hat{\mathbf{A}}\hat{\mathbf{A}}^{\mathrm{T}}||_F^2$, where $\mathbf{A} = (\boldsymbol{\alpha}_1\ \boldsymbol{\alpha}_2)$. EE, estimation error, defined as $||\mathbf{A}\mathbf{A}^T - \hat{\mathbf{A}}\hat{\mathbf{A}}^{\mathrm{T}}||_F^2$. cPVE, proportions of cumulative variation explained. $\cdot(\cdot)$, mean(std).

Method	RE	EE	Sensitivity		Specificity		cPVE	
			1stPC	2ndPC	1stPC	2ndPC	1stPC	2ndPC
**P = 500**								
PCA	31 (0.9)	1.1 (3e-2)	1.0	1.0	0.0	0.0	4.3e-2 (2e-3)	8.2e-2 (2e-3)
SPCA	35 (2)	1.3 (9e-2)	0.49	0.50	0.95	1.0	1.9e-2 (3e-3)	3.9e-2 (4e-3)
SPC	15 (7)	0.54 (3e-1)	0.57	0.60	0.98	1.0	2.8e-2 (3e-3)	5.6e-2 (5e-3)
biological information correctly specified								
Fused sPCA	26 (4)	0.92 (0.2)	1	1	0.7	0.7	3e-2 (3e-3)	5.4e-2 (5e-3)
Grouped sPCA	7.8 (5)	0.28 (0.2)	0.8	0.8	0.97	1	3.2e-2 (2e-3)	6e-2 (3e-3)
biological information correctly specified and 170 extra noise edges are added								
Fused sPCA	33 (10)	1.2 (0.4)	0.94	0.9	0.51	0.5	2.5e-2 (1e-2)	4.4e-2 (2e-2)
Grouped sPCA	8.7 (6)	0.32 (0.2)	0.81	0.8	0.97	1	3.2e-2 (2e-3)	5.9e-2 (3e-3)

Table S5: Simulation results of Setting 2 when extra noise edges are added to structural information. Cumulative proportions of variance explained by true PCs are 0.03 for PC 1 and 0.06 for PC 1 and 2. P, number of variables. RE, reconstruction error, defined as $||\mathbf{X}_{test}\mathbf{A}\mathbf{A}^T - \mathbf{X}_{test}\hat{\mathbf{A}}\hat{\mathbf{A}}^{\mathrm{T}}||_F^2$, where $\mathbf{A} = (\boldsymbol{\alpha}_1\ \boldsymbol{\alpha}_2)$. EE, estimation error, defined as $||\mathbf{A}\mathbf{A}^T - \hat{\mathbf{A}}\hat{\mathbf{A}}^{\mathrm{T}}||_F^2$. cPVE, proportions of cumulative variation explained. $\cdot(\cdot)$, mean(std).

Method	RE	EE	Sensitivity		Specificity		cPVE	
			1stPC	2ndPC	1stPC	2ndPC	1stPC	2ndPC
**P = 500**								
PCA	31 (0.9)	1.1 (3e-2)	1.0	1.0	0.0	0.0	4.3e-2 (2e-3)	8.2e-2 (2e-3)
SPCA	35 (2)	1.3 (9e-2)	0.49	0.50	0.95	1.0	1.9e-2 (3e-3)	3.9e-2 (4e-3)
SPC	15 (7)	0.54 (3e-1)	0.57	0.60	0.98	1.0	2.8e-2 (3e-3)	5.6e-2 (5e-3)
biological information correctly specified								
Fused sPCA	26 (4)	0.92 (0.2)	1	1	0.7	0.7	3e-2 (3e-3)	5.4e-2 (5e-3)
Grouped sPCA	7.8 (5)	0.28 (0.2)	0.8	0.8	0.97	1	3.2e-2 (2e-3)	6e-2 (3e-3)
biological information correctly specified and 170 extra noise edges are added								
Fused sPCA	37 (10)	1.3 (0.5)	0.91	0.8	0.48	0.5	2.3e-2 (1e-2)	4.1e-2 (2e-2)
Grouped sPCA	8.5 (5)	0.31 (0.2)	0.8	0.8	0.97	1	3.2e-2 (2e-3)	6e-2 (3e-3)

Table S6: Prediction accuracy using the PCs of PCA-based methods. $\cdot(\cdot)$ represents $mean(sd)$.

Explained at least 60% of variation				
Fused sPCA	Grouped sPCA	PCA	SPC	SPCA
0.8595 (0.04)	0.8357 (0.05)	0.8395 (0.04)	-	-
Explained at least 50% of variation				
Fused sPCA	Grouped sPCA	PCA	SPC	SPCA
0.8543 (0.04)	0.8407 (0.06)	0.8395 (0.04)	0.8382 (0.04)	-
Explained at least 40% of variation				
Fused sPCA	Grouped sPCA	PCA	SPC	SPCA
0.8322 (0.04)	0.8104 (0.06)	0.8395 (0.04)	0.8643 (0.04)	-
Explained at least 30% of variation				
Fused sPCA	Grouped sPCA	PCA	SPC	SPCA
0.9084 (0.04)	0.7900 (0.05)	0.8395 (0.04)	0.8950 (0.03)	0.7823 (0.04)
